# Supplementary material for: A long-read sequencing strategy with overlapping linkers on adjacent fragments (OLAF-Seq) for targeted resequencing and enrichment
Source: Sci Rep. 2024 Mar 7;14:5583. doi: 10.1038/s41598-024-56402-w (PMC10917763; doi:10.1038/s41598-024-56402-w)
Supplement: Supplementary file 1 — Supplementary Information. [file 41598_2024_56402_MOESM1_ESM.pdf]

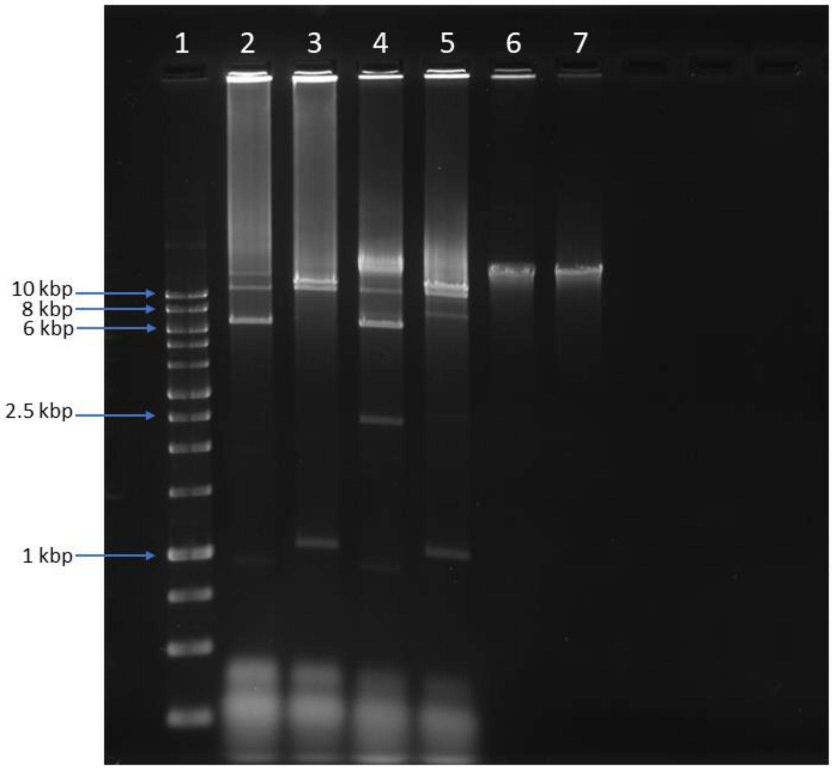


Supplementary Figure 1. Gel image containing Lambda DNA linked-fragments. Lane 1 has a 10 kbp ladder. Lanes 2, 3 and 5 have digestion products from a different sgRNA design. Lane 4 shows the sequenced product following protocol and the sgRNAs designed for Lambda linker generation. Lanes 6 and 7 contain replicates of Lambda DNA as temperature controls without any enzymes or sgRNAs.

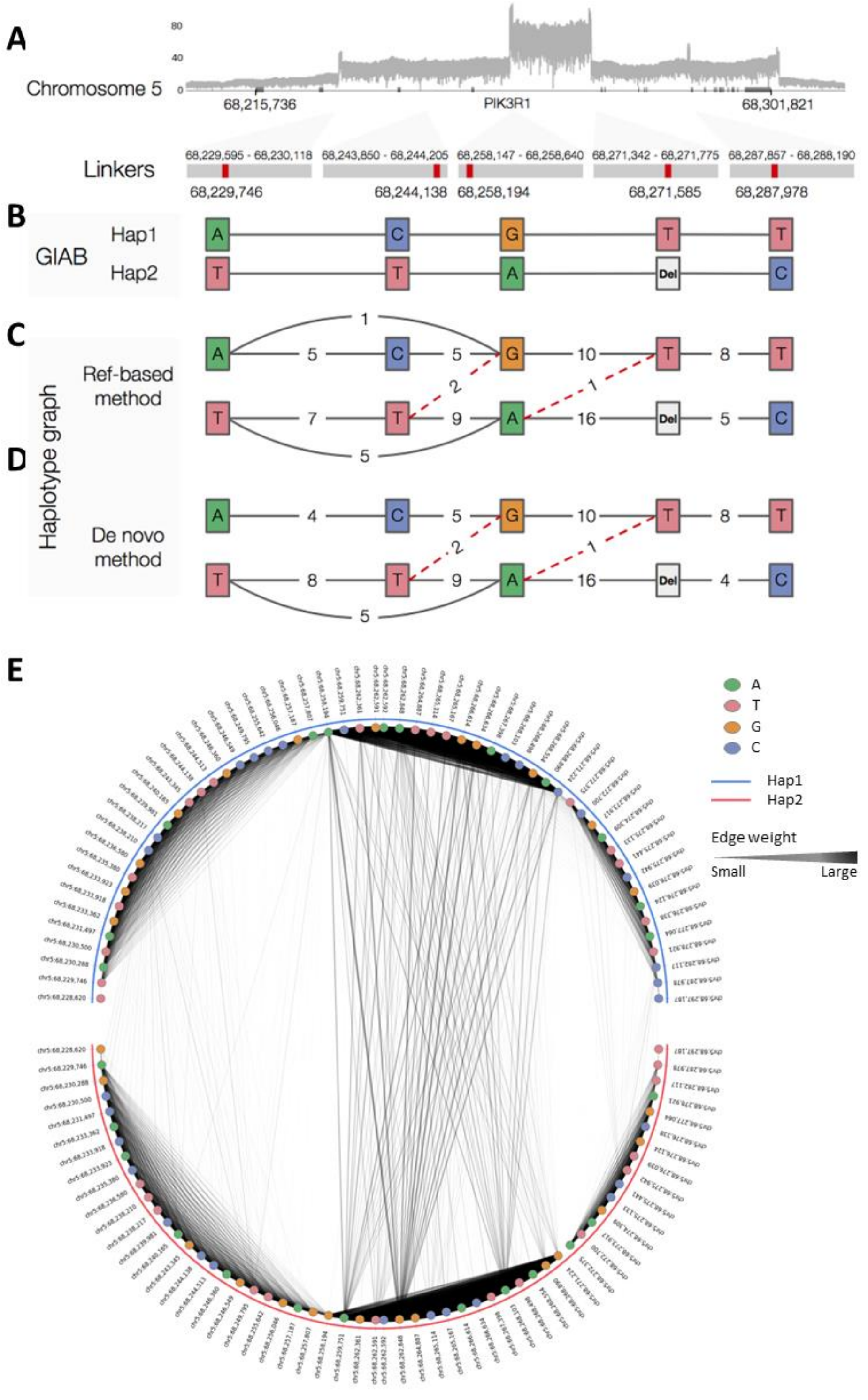


Supplementary Figure 2. Haplotype phasing of the heterozygous variants within the linker regions of the gene PIK3R1. (A) Depth of sequencing reads aligned to the gene region. (B) The six heterozygous SNVs within the four linkers and the corresponding haplotypes phased by GIAB. (C-D) Haplotype graphs constructed (C) by aligning the sequencing reads to the reference genome and (D) by multiple sequence alignment of the sequencing reads. Each solid black line indicates two alleles found on the same read that are from the same GIAB haplotype, and each dotted red line indicates two alleles found on the same read that are from different GIAB haplotypes. Each of these two types of lines is labeled by the number of supporting reads. (E) Haplotype phasing of all heterozygous SNVs within the genes. In each panel, the variant sites are ordered from left to right according to their genomic locations. The two alleles from each variant site are placed at either the upper or lower semi-circle, according to the haplotype that it was assigned to by our method. Both the width and darkness of each edge reflect the number of reads in which the two alleles that define the edge co-appear.

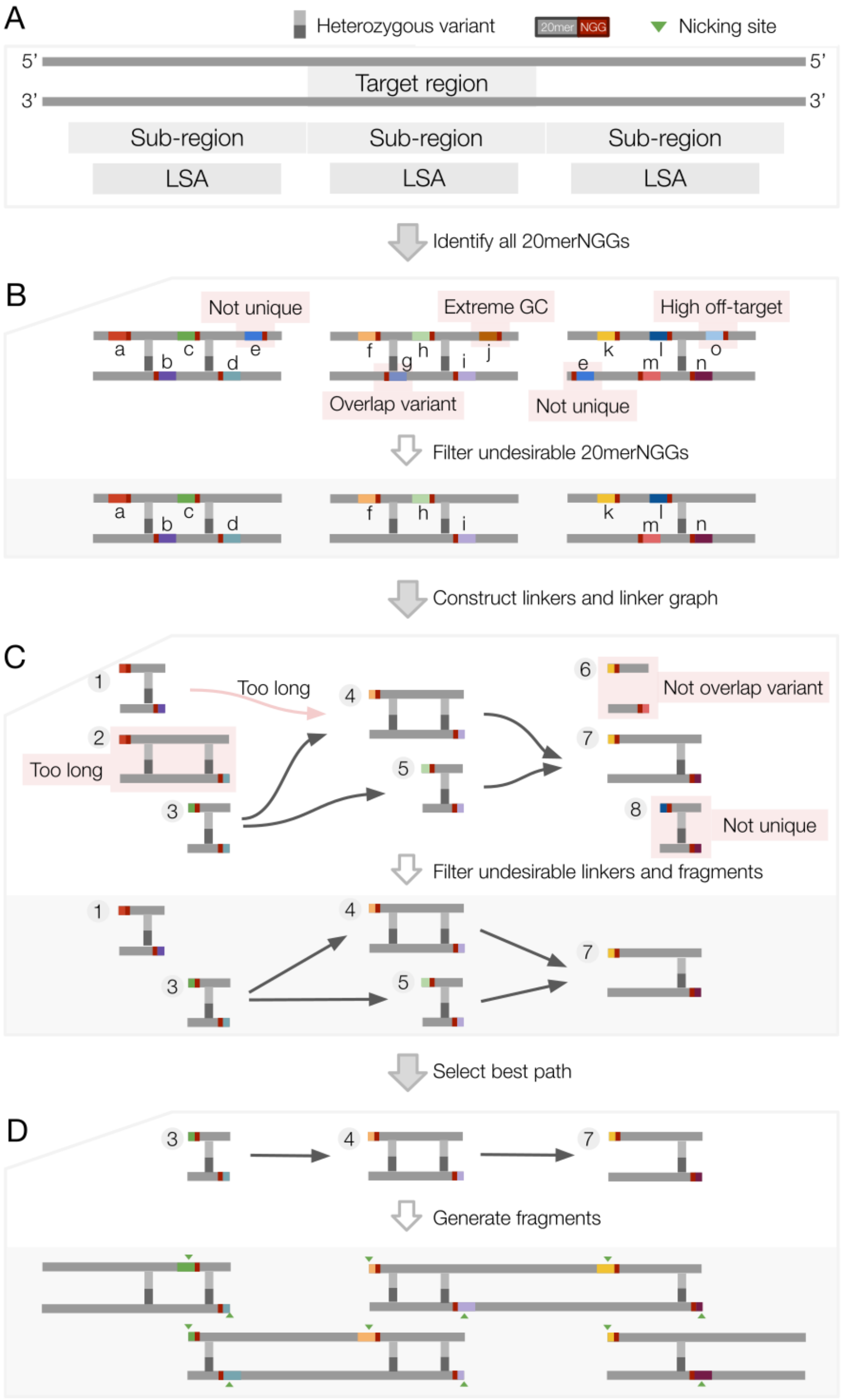


Supplementary Figure 3. The basic workflow of sgRNA design method. (A) In order to cover target regions, we first define a list of sub-regions and further define LSAs within them. (B_ The 20merNGGs (a-o) within the LSAs are collected. The ones that are not unique in the genome (e), overlapping a genetic variant (g), having an extreme GC content (j) or having a high off-target score (o) are removed. (C) For the remaining 20merNGGs, each pair of them from the same LSA located on opposite strands form a candidate linker. The candidate linkers with the two 20merNGGs too close or too far away from each other (2), not overlapping any heterozygous variants (6) or not unique in the genome (8) are removed. For the remaining candidate linkers, a graph is formed by connecting linkers that are located within a suitable range of distance from each other. Edges are not drawn for linker pairs that are not (e.g., 1 or 4). (D) Finally, the best path that starts from a node within the first LSA to a node within the last LSA is chosen. The corresponding DNA fragments can then be inferred from the linkers on this path.

*Supplementary Table 1: Lambda OLAF-Seq guideRNAs*

| Target Genome | sgRNA ID | sgRNA sequence | start position | gRNA strand |
|---|---|---|---|---|
| Lambda | sgRNA1 | AGCGCCATGTTTACCAGTCC | 6270 | + |
| Lambda | sgRNA2 | TCCACCTCTGAATTTACGCC | 6339 | - |
| Lambda | sgRNA3 | AGGTCTTCATCGTCCACCTC | 13090 | + |
| Lambda | sgRNA4 | TTCGGTCCCTTCTGTAAGAA | 13144 | - |
| Lambda | sgRNA5 | TGAATGACTTCCCCAATTAT | 24569 | + |
| Lambda | sgRNA6 | CTGTAGCTGCTGAAACGTTG | 24629 | - |
| Lambda | sgRNA7 | CCATGCAACATGAATAACAG | 27154 | + |
| Lambda | sgRNA8 | CGCCGAACGATTAGCTCTTC | 27221 | - |
| Lambda | sgRNA9 | CGACTATTGATAAAAATCAA | 34261 | + |
| Lambda | sgRNA10 | CAGTTTGATGAGTATAGAAA | 34478 | - |
| Lambda | sgRNA11 | GAAGGTTTTACCAATGGCTC | 47446 | + |
| Lambda | sgRNA12 | ATGTTTTCACTTAATAGTAT | 47556 | - |
| Lambda | sgRNA13 | CTGTAGCTGCTGAAACGTTG | 24629 | + |
| Lambda | sgRNA14 | ACAGGTATCGTTTGGAGGCA | 24722 | - |
| Lambda | sgRNA15 | AGTTACCCCTCTAAGTAATG | 26560 | + |
| Lambda | sgRNA16 | CCATGCAACATGAATAACAG | 27154 | - |
| Lambda | sgRNA17 | TCAGAATGAGTGAACAACCA | 21980 | + |
| Lambda | sgRNA18 | AAGCAGATAAATATATTTTT | 23004 | - |
| Lambda | sgRNA19 | CTGTAGCTGCTGAAACGTTG | 24629 | + |
| Lambda | sgRNA20 | ACAGGTATCGTTTGGAGGCA | 24719 | - |

*Supplementary Table 2. Cancer genes loci in the human genome targeted with OLAF-Seq*

| Gene name | Chromosome | Start position | End position | Length | Category |
|---|---|---|---|---|---|
| ARFRP1 | chr20 | 63698641 | 63708013 | 9372 | Short |
| B2M | chr15 | 44711486 | 44718159 | 6673 | Short |
| BAP1 | chr3 | 52401003 | 52410105 | 9102 | Short |
| BCL2A1 | chr15 | 79960889 | 79971301 | 10412 | Short |
| BCL2L2 | chr14 | 23306761 | 23311759 | 4998 | Short |
| BRCA2 | chr13 | 32315479 | 32399672 | 84193 | Long |
| BTG1 | chr12 | 92140277 | 92145897 | 5620 | Short |
| CCND1 | chr11 | 69641104 | 69654474 | 13370 | Short |
| CCNE1 | chr19 | 29811993 | 29824317 | 12324 | Short |
| CD79A | chr19 | 41877119 | 41881372 | 4253 | Short |
| CD79B | chr17 | 63928735 | 63932354 | 3619 | Short |
| CDKN1A | chr6 | 36676459 | 36687339 | 10880 | Short |
| CDKN1B | chr12 | 12717269 | 12722383 | 5114 | Short |
| CDKN2B | chr9 | 22002902 | 22009313 | 6411 | Short |
| CDKN2C | chr1 | 50968694 | 50974637 | 5943 | Short |
| CIC | chr19 | 42268536 | 42295796 | 27260 | Long |
| CNOT3 | chr19 | 54137688 | 54155708 | 18020 | Short |
| COL2A1 | chr12 | 47972964 | 48006212 | 33248 | Long |
| CRCT1 | chr1 | 152514501 | 152516010 | 1509 | Short |
| CRKL | chr22 | 20917406 | 20953749 | 36343 | Long |
| CSF3R | chr1 | 36466042 | 36483314 | 17272 | Short |
| CTNNB1 | chr3 | 41199421 | 41240453 | 41032 | Long |
| CXCR4 | chr2 | 136114348 | 136118155 | 3807 | Short |
| DAXX | chr6 | 33318557 | 33323016 | 4459 | Short |
| DDX41 | chr5 | 177511576 | 177517326 | 5750 | Short |
| DIS3 | chr13 | 72755401 | 72782128 | 26727 | Long |
| DUSP4 | chr8 | 29333061 | 29350750 | 17689 | Short |
| EME2 | chr16 | 1773221 | 1776714 | 3493 | Short |
| EPCAM | chr2 | 47369147 | 47387028 | 17881 | Short |
| EPHA2 | chr1 | 16124336 | 16156109 | 31773 | Long |
| EPOR | chr19 | 11377204 | 11384342 | 7138 | Short |
| ERBB3 | chr12 | 56080024 | 56103507 | 23483 | Long |
| ERRFI1 | chr1 | 8011718 | 8026333 | 14615 | Short |
| ESPL1 | chr12 | 53268256 | 53293643 | 25387 | Long |
| FAM46C | chr1 | 117605981 | 117628389 | 22408 | Long |
| FANCE | chr6 | 35452338 | 35467106 | 14768 | Short |
| FANCG | chr9 | 35073837 | 35080016 | 6179 | Short |
| FGF3 | chr11 | 69809967 | 69819424 | 9457 | Short |
| FGF4 | chr11 | 69771015 | 69775403 | 4388 | Short |
| FGF6 | chr12 | 4434141 | 4445799 | 11658 | Short |

| | | | | | |
|---|---|---|---|---|---|
| FGFR3 | chr4 | 1793298 | 1808872 | 15574 | Short |
| FOXA1 | chr14 | 37589551 | 37595120 | 5569 | Short |
| FUS | chr16 | 31180109 | 31194871 | 14762 | Short |
| GATA1 | chrX | 48786539 | 48794311 | 7772 | Short |
| GATA2 | chr3 | 128479421 | 128493187 | 13766 | Short |
| GATA3 | chr10 | 8045419 | 8075201 | 29782 | Long |
| GLI1 | chr12 | 57460134 | 57472451 | 12317 | Short |
| H3F3B | chr17 | 75776433 | 75779935 | 3502 | Short |
| HNF1A | chr12 | 120977682 | 121002512 | 24830 | Long |
| HOXB13 | chr17 | 48724762 | 48728749 | 3987 | Short |
| HRAS | chr11 | 532241 | 535567 | 3326 | Short |
| HSP90AB1 | chr6 | 44246165 | 44253888 | 7723 | Short |
| HSPA5 | chr9 | 125234847 | 125241387 | 6540 | Short |
| IDH2 | chr15 | 90083977 | 90102554 | 18577 | Short |
| IGF2 | chr11 | 2129111 | 2149603 | 20492 | Long |
| IKBKE | chr1 | 206470242 | 206496890 | 26648 | Long |
| KNSTRN | chr15 | 40382720 | 40394288 | 11568 | Short |
| LZTR1 | chr22 | 20982268 | 20999037 | 16769 | Short |
| MALAT1 | chr11 | 65497737 | 65506516 | 8779 | Short |
| MAP3K1 | chr5 | 56815072 | 56896152 | 81080 | Long |
| MCL1 | chr1 | 150574550 | 150579738 | 5188 | Short |
| MEN1 | chr11 | 64803513 | 64811294 | 7781 | Short |
| MPL | chr1 | 43336874 | 43354464 | 17590 | Short |
| MUTYH | chr1 | 45329241 | 45340470 | 11229 | Short |
| MYC | chr8 | 127735433 | 127742951 | 7518 | Short |
| MYCL | chr1 | 39895423 | 39902015 | 6592 | Short |
| MYCN | chr2 | 15940437 | 15947007 | 6570 | Short |
| MYD88 | chr3 | 38137438 | 38143022 | 5584 | Short |
| NBN | chr8 | 89933335 | 89984724 | 51389 | Long |
| NF2 | chr22 | 29603555 | 29698600 | 95045 | Long |
| NFKBIE | chr6 | 44258165 | 44265788 | 7623 | Short |
| NKX2-1 | chr14 | 36516396 | 36520225 | 3829 | Short |
| NOTCH1 | chr9 | 136494432 | 136545786 | 51354 | Long |
| NRAS | chr1 | 114704463 | 114716894 | 12431 | Short |
| OR5L1 | chr11 | 55811366 | 55812476 | 1110 | Short |
| PHOX2B | chr4 | 41744081 | 41748970 | 4889 | Short |
| PIK3R1 | chr5 | 68215736 | 68301821 | 86085 | Long |
| PIK3R2 | chr19 | 18153177 | 18170533 | 17356 | Short |
| POLD1 | chr19 | 50384290 | 50418018 | 33728 | Long |
| POLR2L | chr11 | 839720 | 842529 | 2809 | Short |
| PTCH2 | chr1 | 44819843 | 44842944 | 23101 | Long |
| RAD51C | chr17 | 58692139 | 58734342 | 42203 | Long |

| | | | | | |
|---|---|---|---|---|---|
| RIT1 | chr1 | 155897807 | 155911402 | 13595 | Short |
| RNF43 | chr17 | 58352499 | 58418894 | 66395 | Long |
| RPL10 | chrX | 154398064 | 154402339 | 4275 | Short |
| RRAS | chr19 | 49635291 | 49640143 | 4852 | Short |
| SDHD | chr11 | 112086823 | 112095801 | 8978 | Short |
| SH2B3 | chr12 | 111405106 | 111451623 | 46517 | Long |
| SHH | chr7 | 155799983 | 155812273 | 12290 | Short |
| SMARCA4 | chr19 | 10960921 | 11062282 | 1E+05 | Long |
| SMARCB1 | chr22 | 23786930 | 23834518 | 47588 | Long |
| SOX2 | chr3 | 181711923 | 181714436 | 2513 | Short |
| SPRY1 | chr4 | 123396790 | 123403760 | 6970 | Short |
| SPRY2 | chr13 | 80335975 | 80341115 | 5140 | Short |
| SPRY4 | chr5 | 142310426 | 142325055 | 14629 | Short |
| STK11 | chr19 | 1205798 | 1228435 | 22637 | Long |
| TBX3 | chr12 | 114670253 | 114684164 | 13911 | Short |
| TFE3 | chrX | 49028725 | 49043517 | 14792 | Short |
| TMPRSS2 | chr21 | 41464550 | 41508158 | 43608 | Long |
| TP53 | chr17 | 7668401 | 7687550 | 19149 | Long |
| VEGFA | chr6 | 43770208 | 43786487 | 16279 | Short |
| VHL | chr3 | 10141634 | 10153670 | 12036 | Short |
| ZNF703 | chr8 | 37695750 | 37700021 | 4271 | Short |

*Supplementary Table 3: guideRNAs designed for targeted sequencing of NA12878 genome*

| Gene | gRNA ID | gRNA sequence | gRNA chromosome | gRNA start position | gRNA strand |
|---|---|---|---|---|---|
| ARFRP1 | ARFRP1+1 | TGCCCAGGACGGTGTCTTCG | chr20 | 63696239 | + |
| ARFRP1 | ARFRP1+2 | GGAAGTCCAAGTCCGATGTG | chr20 | 63710748 | + |
| B2M | B2M+1 | TTAACACTGATTACTCATAG | chr15 | 44706493 | + |
| B2M | B2M+2 | TGTCAGATCCCGCTGGTTGA | chr15 | 44720639 | + |
| BAP1 | BAP1+1 | ACCCAGCACAGACCGTGACC | chr3 | 52395900 | + |
| BAP1 | BAP1+2 | GGTCGGTTTCTACTAACGGT | chr3 | 52410577 | + |
| BCL2A1 | BCL2A1+1 | ACCTGAAAGGGTCTACCGTG | chr15 | 79960749 | + |
| BCL2A1 | BCL2A1+2 | GTGAAAGGACTGGTGCGGGG | chr15 | 79975903 | + |
| BCL2L2 | BCL2L2+1 | TGGTGTCTGCTAGCCCCATA | chr14 | 23306413 | + |
| BCL2L2 | BCL2L2+2 | CGGTGAGGCGATCGGAAGAT | chr14 | 23321098 | + |
| BRCA2 | BRCA2+1 | AATGCAAAGTTAGCCGTGCG | chr13 | 32314693 | + |
| BRCA2 | BRCA2-1 | TCACTTAGTGGTAGTGGGCG | chr13 | 32315054 | - |
| BRCA2 | BRCA2+2 | CCACCTTGTGAGTAGTACTA | chr13 | 32329230 | + |
| BRCA2 | BRCA2-2 | ACCCCATGAATAGGGGACTA | chr13 | 32329721 | - |
| BRCA2 | BRCA2+3 | TGAGCCACTGTGCCTAATCA | chr13 | 32344760 | + |
| BRCA2 | BRCA2-3 | AGTGACAATACTTATTGCCG | chr13 | 32345132 | - |
| BRCA2 | BRCA2+4 | ACAAGTCATCGGCATATAGA | chr13 | 32361109 | + |
| BRCA2 | BRCA2-4 | TGCCTACAAGGCCAACGATC | chr13 | 32361635 | - |
| BRCA2 | BRCA2+5 | TTTCCTGGAAAACTTATAGC | chr13 | 32376219 | + |
| BRCA2 | BRCA2-5 | TGTCTTGTTAGTGCACGTGA | chr13 | 32376714 | - |
| BRCA2 | BRCA2+6 | CACTATGCCCAGCGTGTCTT | chr13 | 32388404 | + |
| BRCA2 | BRCA2-6 | ACTCACTTTAAATTCGACAA | chr13 | 32388805 | - |
| BRCA2 | BRCA2+7 | CTCATTTGTAAATCCACCGT | chr13 | 32402983 | + |
| BRCA2 | BRCA2-7 | TCTTGAAGCACGTAATTGAG | chr13 | 32403473 | - |
| BTG1 | BTG1+1 | AAAAAATCAATCTAGCCGTT | chr12 | 92136055 | + |
| BTG1 | BTG1+2 | AGATGAGTAGAGATAGTAGT | chr12 | 92150192 | + |
| CCND1 | CCND1+1 | TTAGTAATCCTAATTCCCGG | chr11 | 69639581 | + |
| CCND1 | CCND1+2 | AGGCTGGTGGCAAGTGCACG | chr11 | 69654274 | + |
| CCNE1 | CCNE1+1 | TTTGCCGCTCCAGCGCCGCT | chr19 | 29811607 | + |
| CCNE1 | CCNE1+2 | CAAATTTGAAAGTACGTTTG | chr19 | 29828817 | + |
| CD79A | CD79A+1 | CAAGGAAGAGCTAGCCGGGG | chr19 | 41868851 | + |
| CD79A | CD79A+2 | ATCACTACCATTGTACGGGT | chr19 | 41883559 | + |
| CD79B | CD79B+1 | ACGCCGAAAGATGCTCTATC | chr17 | 63919667 | + |
| CD79B | CD79B+2 | ACACACACATCCCGTGGACA | chr17 | 63934327 | + |
| CDKN1A | CDKN1A+1 | ATGCTGCTCCACCGCACTCT | chr6 | 36676302 | + |
| CDKN1A | CDKN1A+2 | TAAGACCTGACCCTAATTAC | chr6 | 36690896 | + |
| CDKN1B | CDKN1B+1 | GAAGAGTCTTTGGTTGCTCG | chr12 | 12709297 | + |
| CDKN1B | CDKN1B+2 | GCTGGTGCCTCGGGACGAGA | chr12 | 12723947 | + |
| CDKN2B | CDKN2B+1 | GGACCTCCAAGATCTCGGAA | chr9 | 21994542 | + |
| CDKN2B | CDKN2B+2 | TCAAAGCCGCTCTGGCCGCA | chr9 | 22009233 | + |

| | | | | | |
|---|---|---|---|---|---|
| CDKN2C | CDKN2C+1 | CCACTGCAGCGTTCAAACGC | chr1 | 50959670 | + |
| CDKN2C | CDKN2C+2 | AATCTTCAATAAACGTGGGG | chr1 | 50974258 | + |
| CIC | CIC+1 | GCCGCTGCTGATTGGCTGCG | chr19 | 42268473 | + |
| CIC | CIC-1 | TGGCCCGAACGTTTGTTGAC | chr19 | 42268817 | - |
| CIC | CIC+2 | CAGGGCTCAGGAAGCGAGTG | chr19 | 42285129 | + |
| CIC | CIC-2 | GGGAGCTCTGTGCGAGGCAC | chr19 | 42285565 | - |
| CIC | CIC+3 | AGCACCACAACCCGGGCCTG | chr19 | 42299970 | + |
| CIC | CIC-3 | AATGGCATTTGGTTAACACC | chr19 | 42300385 | - |
| CNOT3 | CNOT3+1 | ATCACCCAATCCGCGAAAGG | chr19 | 54137587 | + |
| CNOT3 | CNOT3+2 | CACCTTTGAGTACCGCTACC | chr19 | 54155364 | + |
| COL2A1 | COL2A1+1 | TCCCACGTCTCCGATCCAGC | chr12 | 47972596 | + |
| COL2A1 | COL2A1-1 | CCATTAATCCTCCGTTCTGT | chr12 | 47972930 | - |
| COL2A1 | COL2A1+2 | GGGTCTCCTTCACCCGGCTC | chr12 | 47988541 | + |
| COL2A1 | COL2A1-2 | CCAACAGCTGAGCAAGCTCG | chr12 | 47988888 | - |
| COL2A1 | COL2A1+3 | ACTGGAATTAGACCTATATG | chr12 | 48006996 | + |
| COL2A1 | COL2A1-3 | CTGTAGATCGGGATATTCCC | chr12 | 48007450 | - |
| CRCT1 | CRCT1+1 | CCATCAGAGGGGAGGCGTAA | chr1 | 152502685 | + |
| CRCT1 | CRCT1+2 | GGAAGAGACACTCGGGTCTC | chr1 | 152517212 | + |
| CRKL | CRKL+1 | TAGCTGTACCTAAACCTATT | chr22 | 20908188 | + |
| CRKL | CRKL-1 | TTTGATACAAGAACGTGCAT | chr22 | 20908684 | - |
| CRKL | CRKL+2 | CTGTGGAACTCCTACTGTAG | chr22 | 20921443 | + |
| CRKL | CRKL-2 | AATTTGCCCGGCATGGTTGC | chr22 | 20921876 | - |
| CRKL | CRKL+3 | AAAAGACAGTGAGGTTGATC | chr22 | 20940235 | + |
| CRKL | CRKL-3 | GATGTAATCCAAATTACTAC | chr22 | 20940755 | - |
| CRKL | CRKL+4 | GACAGGATCGTTGTCCCTAC | chr22 | 20953354 | + |
| CRKL | CRKL-4 | TGAACTGCCAGGAGGCTAGT | chr22 | 20953801 | - |
| CSF3R | CSF3R+1 | TAACCTATGTGGGCCCTGTA | chr1 | 36464863 | + |
| CSF3R | CSF3R+2 | GGCAAGACAGGAGGACGTCT | chr1 | 36483433 | + |
| CTNNB1 | CTNNB1+1 | TGCAGACCACAGCGCCCTCA | chr3 | 41199225 | + |
| CTNNB1 | CTNNB1-1 | GTCCGACCGTCCTCGACCTG | chr3 | 41199638 | - |
| CTNNB1 | CTNNB1+2 | TGCTTAATCAGTTCATCGAT | chr3 | 41218025 | + |
| CTNNB1 | CTNNB1-2 | AGATACATACGAGAGTAAAT | chr3 | 41218451 | - |
| CTNNB1 | CTNNB1+3 | CAGGGAGGTCTGTAGATAGC | chr3 | 41230362 | + |
| CTNNB1 | CTNNB1-3 | GCCAAAAGACTGGATATCCC | chr3 | 41230828 | - |
| CTNNB1 | CTNNB1+4 | TCCCCAGACAGGCAACACCG | chr3 | 41244830 | + |
| CTNNB1 | CTNNB1-4 | ACTTAGTGCCCACGGTGAGG | chr3 | 41245275 | - |
| CXCR4 | CXCR4+1 | TTTTATTTCCTCTTTTGCGA | chr2 | 136106742 | + |
| CXCR4 | CXCR4+2 | TGGCCTGTGACCCCGGAATG | chr2 | 136121312 | + |
| DAXX | DAXX+1 | TTCCTCCAAAGGGCGGCATG | chr6 | 33314095 | + |
| DAXX | DAXX+2 | CAGATACCTGAGGTCGGAGT | chr6 | 33328662 | + |
| DDX41 | DDX41+1 | TTACAGAGCATGTTTGCGTG | chr5 | 177502376 | + |
| DDX41 | DDX41+2 | GGAGTTCCGGATCGCGCAGA | chr5 | 177517047 | + |

| | | | | | |
|---|---|---|---|---|---|
| DIS3 | DIS3+1 | CATAATTTCAGGCACTAGTC | chr13 | 72754101 | + |
| DIS3 | DIS3-1 | AGAAACTGGCATATAACAGC | chr13 | 72754490 | - |
| DIS3 | DIS3+2 | TACTTTGGCTTGGTACTAGC | chr13 | 72770514 | + |
| DIS3 | DIS3-2 | TAATTCAAAGGTTCGTTTCA | chr13 | 72770912 | - |
| DIS3 | DIS3+3 | GAGTGATTTAAACGTAGATA | chr13 | 72784794 | + |
| DIS3 | DIS3-3 | AACAAACAAATTCAGTCCGA | chr13 | 72785298 | - |
| DUSP4 | DUSP4+1 | ATCTCCACTGCGCTATTAAG | chr8 | 29332947 | + |
| DUSP4 | DUSP4+2 | GGAGAGTGTGTTTACGAGAG | chr8 | 29350537 | + |
| EME2 | EME2+1 | GTGTACGTGGGTTCACGGGG | chr16 | 1767961 | + |
| EME2 | EME2+2 | GTGACAAAGCTATTGCCGAG | chr16 | 1782657 | + |
| EPCAM | EPCAM+1 | AAAGATCCCTAACGCCGCCA | chr2 | 47369056 | + |
| EPCAM | EPCAM+2 | CTGCAGGCCCAGAAGCGTCC | chr2 | 47388318 | + |
| EPHA2 | EPHA2+1 | GATCTATTTCGACAGCTCCG | chr1 | 16124004 | + |
| EPHA2 | EPHA2-1 | CAGATGATTCAAACCGATCT | chr1 | 16124352 | - |
| EPHA2 | EPHA2+2 | TCCATGCAGACTAGCCTGTC | chr1 | 16138810 | + |
| EPHA2 | EPHA2-2 | AGGAAGAAGCCCCCGGAGTC | chr1 | 16139185 | - |
| EPHA2 | EPHA2+3 | TCAAGGAGCGCCGGGCTCTA | chr1 | 16155777 | + |
| EPHA2 | EPHA2-3 | GGCGTTGGTGACGTCACGCA | chr1 | 16156127 | - |
| EPOR | EPOR+1 | AGCTCTGAACTGGTTCATCC | chr19 | 11375396 | + |
| EPOR | EPOR+2 | ATGACACAAATTGGCCCAAT | chr19 | 11390573 | + |
| ERBB3 | ERBB3+1 | GAATGCATAGCTCAAACGGG | chr12 | 56068709 | + |
| ERBB3 | ERBB3-1 | AATTGGCCGGGCGCGGTGTC | chr12 | 56069234 | - |
| ERBB3 | ERBB3+2 | GGTGTTCTGTGGATAGTGCA | chr12 | 56084124 | + |
| ERBB3 | ERBB3-2 | GGGTCTCGCTCTGTTACGAG | chr12 | 56084588 | - |
| ERBB3 | ERBB3+3 | GATCTTCACTCCTTATCCGA | chr12 | 56103271 | + |
| ERBB3 | ERBB3-3 | ATGAAAAGGACTATGCGCTA | chr12 | 56103678 | - |
| ERRFI1 | ERRFI1+1 | TGCCCGATGAAGGTCGTGGA | chr1 | 8011107 | + |
| ERRFI1 | ERRFI1+2 | ATCCCAGAGACTCCGCGTCG | chr1 | 8025997 | + |
| ESPL1 | ESPL1+1 | AAGGTGAGCGGATGACTCAA | chr12 | 53262845 | + |
| ESPL1 | ESPL1-1 | TGCACAGGTGAACTTACGTG | chr12 | 53263274 | - |
| ESPL1 | ESPL1+2 | TCCAGGGTATCGAGCGGGAT | chr12 | 53277464 | + |
| ESPL1 | ESPL1-2 | GCTGGAGACACCGTACAGCT | chr12 | 53277906 | - |
| ESPL1 | ESPL1+3 | CCGCGACATTGACCGCTACA | chr12 | 53293308 | + |
| ESPL1 | ESPL1-3 | TCAGATAAACTACGCAAAAC | chr12 | 53293661 | - |
| FAM46C | FAM46C+1 | GGGTTGGAAGAGTAGGGCGG | chr1 | 117604869 | + |
| FAM46C | FAM46C-1 | TCCATTCATAACATAGCGAG | chr1 | 117605276 | - |
| FAM46C | FAM46C+2 | TGGATAAAAATGATACGTTG | chr1 | 117619307 | + |
| FAM46C | FAM46C-2 | TGCCAAACTAAGATCTAGAT | chr1 | 117619707 | - |
| FAM46C | FAM46C+3 | AATGAAAGGTACTGCTCCGG | chr1 | 117633858 | + |
| FAM46C | FAM46C-3 | TAATTGGTTTCCACCGTGGT | chr1 | 117634350 | - |
| FANCE | FANCE+1 | GAGAGGGTTGGGGTCGTCAG | chr6 | 35452088 | + |
| FANCE | FANCE+2 | CAGGGACAAACTTTCCGATA | chr6 | 35466911 | + |

| | | | | | |
|---|---|---|---|---|---|
| FANCG | FANCG+1 | GTAATCATAAAATCGGATAC | chr9 | 35065208 | + |
| FANCG | FANCG+2 | GTCTGACTGGGGCAGTCGCA | chr9 | 35079868 | + |
| FGF3 | FGF3+1 | AGAACGTGAAATGGCGAGAT | chr11 | 69806930 | + |
| FGF3 | FGF3+2 | AAAGGTGACCCCTCTATAGG | chr11 | 69821429 | + |
| FGF4 | FGF4+1 | TTATTGTTATTTCCATCCCG | chr11 | 69760397 | + |
| FGF4 | FGF4+2 | AGCAAGGCCAGCAGGACCGC | chr11 | 69775025 | + |
| FGF6 | FGF6+1 | GATTTTAGCCCTCGTTTGAA | chr12 | 4434025 | + |
| FGF6 | FGF6+2 | TTTTATCGGGTGATGCGATG | chr12 | 4448658 | + |
| FGFR3 | FGFR3+1 | GAGCCACCGAGTCGGCACAG | chr4 | 1792084 | + |
| FGFR3 | FGFR3+2 | CAGGCAGGGAGACGGTTTCC | chr4 | 1808461 | + |
| FOXA1 | FOXA1+1 | GAAGGTGTGCACTCGTCAAG | chr14 | 37584769 | + |
| FOXA1 | FOXA1+2 | ACAGAGTCCTAACCCGTAAC | chr14 | 37599370 | + |
| FUS | FUS+1 | TCCCCCTGCTCTTCCGCGTT | chr16 | 31180052 | + |
| FUS | FUS+2 | ATGGAGCACTAGGCGTAAAT | chr16 | 31194909 | + |
| GATA1 | GATA1+1 | ACGGGAGCCTAGGTCGAGCG | chrX | 48786349 | + |
| GATA1 | GATA1+2 | GCAGTGGTTACCCGTTGGAG | chrX | 48800901 | + |
| GATA2 | GATA2+1 | TGAGAAGAGATACTATGATC | chr3 | 128478724 | + |
| GATA2 | GATA2+2 | TTTCGACATCACTTTGCGGG | chr3 | 128493228 | + |
| GATA3 | GATA3+1 | AACAGGAGAAATAGTCGGAA | chr10 | 8044864 | + |
| GATA3 | GATA3-1 | AACAGGCAAACCGCACATCA | chr10 | 8045197 | - |
| GATA3 | GATA3+2 | GCATATTCTCTGCGTGGAAA | chr10 | 8059389 | + |
| GATA3 | GATA3-2 | CTCTGAAATGGACACGCCTA | chr10 | 8059898 | - |
| GATA3 | GATA3+3 | CTAAGGTGGTTGTGCTCGGA | chr10 | 8074890 | + |
| GATA3 | GATA3-3 | AGAAGAGCTCGGCCACCACG | chr10 | 8075236 | - |
| GLI1 | GLI1+1 | AACTTCGAGACGTAGAGCCG | chr12 | 57459339 | + |
| GLI1 | GLI1+2 | CCAAGGGCAGCACGGAAATG | chr12 | 57474040 | + |
| H3F3B | H3F3B+1 | TATCTTGCTGATTATCACGT | chr17 | 75771628 | + |
| H3F3B | H3F3B+2 | ATCTGGAAACTTCACGTAAT | chr17 | 75786302 | + |
| HNF1A | HNF1A+1 | GAGAAGTGGGGTCGTCATGT | chr12 | 120970724 | + |
| HNF1A | HNF1A-1 | CCAACACCCTGCAAGCGGCC | chr12 | 120971125 | - |
| HNF1A | HNF1A+2 | TCCTAGGAGCCCCGGAAAGA | chr12 | 120984687 | + |
| HNF1A | HNF1A-2 | ATCTCTTGAGCCCGAGAGGT | chr12 | 120985050 | - |
| HNF1A | HNF1A+3 | CTGCAGCTTGTAGCCAGCCG | chr12 | 121002435 | + |
| HNF1A | HNF1A-3 | CATGGTAGTGAGCGCCTGTG | chr12 | 121002854 | - |
| HOXB13 | HOXB13+1 | TCGAAGCGCCCCACGCCAGA | chr17 | 48719035 | + |
| HOXB13 | HOXB13+2 | CTGAACTGCTTGGGGTCGCC | chr17 | 48733551 | + |
| HRAS | HRAS+1 | CTCTTGTGGGTCAGGGTGCG | chr11 | 529541 | + |
| HRAS | HRAS+2 | GGCTGGGGCGTGCAGTGATC | chr11 | 544168 | + |
| HSP90AB1 | HSP90AB1+1 | CCCGTGCTCCGTGTACGTGG | chr6 | 44246131 | + |
| HSPA5 | HSPA5+1 | TCAAGCTGTGAGGTCCATTA | chr9 | 125229868 | + |
| HSPA5 | HSPA5+2 | GTGCTTCTCAAAGGTTAGAC | chr9 | 125243413 | + |
| IDH2 | IDH2+1 | GTGTCCGAGAACTCCGCAGT | chr15 | 90083790 | + |

| | | | | | |
|---|---|---|---|---|---|
| IDH2 | IDH2+2 | AGCTGGAGAGCGAACGAGCA | chr15 | 90102394 | + |
| IGF2 | IGF2+1 | GTCTCCCCGGCAAGCTAACC | chr11 | 2122958 | + |
| IGF2 | IGF2-1 | CATGGTCCCAAGACGCCTGC | chr11 | 2123375 | - |
| IGF2 | IGF2+2 | ATCCCGTAGCGAAGTGCCAT | chr11 | 2137462 | + |
| IGF2 | IGF2-2 | TGTCTGCAGGGGCGACTTCC | chr11 | 2137966 | - |
| IGF2 | IGF2+3 | AAAGCGGAGATTTCCTAATC | chr11 | 2152106 | + |
| IGF2 | IGF2-3 | TGTGAACTGGGTTAGCCCCC | chr11 | 2152494 | - |
| IKBKE | IKBKE+1 | TTCTGGGAGGGTCTTGCATC | chr1 | 206468844 | + |
| IKBKE | IKBKE-1 | GTTGGTGAGGTGGATTGAAC | chr1 | 206469222 | - |
| IKBKE | IKBKE+2 | TATCTTATTGTGTCGCAGGA | chr1 | 206483188 | + |
| IKBKE | IKBKE-2 | TGTCTCTAGATAGTAGGTTA | chr1 | 206483714 | - |
| IKBKE | IKBKE+3 | TTATTTGGGCCTCAAGCCCG | chr1 | 206497741 | + |
| IKBKE | IKBKE-3 | TTGATCACTACAGTGAGTCG | chr1 | 206498227 | - |
| KNSTRN | KNSTRN+1 | AACACTGTTGGGTAATCAGC | chr15 | 40382362 | + |
| KNSTRN | KNSTRN+2 | TGCACTAACCTTAAGTGTAC | chr15 | 40396961 | + |
| LZTR1 | LZTR1+1 | AGAGCTGGAGGGCGCAAGTG | chr22 | 20982226 | + |
| LZTR1 | LZTR1+2 | TTCATTTGTGCTCCTCGGCA | chr22 | 20999073 | + |
| MALAT1 | MALAT1+1 | TCAGTTTCCTCATCCGTCTA | chr11 | 65492506 | + |
| MALAT1 | MALAT1+2 | TGTCCCTTTGAACGTGCTGT | chr11 | 65507189 | + |
| MAP3K1 | MAP3K1+1 | TTGCAGCAGGCGACGGTCCC | chr5 | 56814937 | + |
| MAP3K1 | MAP3K1-1 | AGTGGGCTGTGCTCTCCGCC | chr5 | 56815349 | - |
| MAP3K1 | MAP3K1+2 | TTGTAAGAAGTTTCTGCGTA | chr5 | 56830986 | + |
| MAP3K1 | MAP3K1-2 | TGGAGTTCACTGGTTTGCGA | chr5 | 56831388 | - |
| MAP3K1 | MAP3K1+3 | TGGCAAAGACAGTATGTCTC | chr5 | 56849679 | + |
| MAP3K1 | MAP3K1-3 | AAAACAATCCTAGTCCATTG | chr5 | 56850160 | - |
| MAP3K1 | MAP3K1+4 | TATTTACACAAGTAGTCCAC | chr5 | 56868229 | + |
| MAP3K1 | MAP3K1-4 | TTTAAGAAACCATTACGGCC | chr5 | 56868698 | - |
| MAP3K1 | MAP3K1+5 | AGGCATGATAGTGTACACCT | chr5 | 56883046 | + |
| MAP3K1 | MAP3K1-5 | TGTTTTGCTTTGGTATGTCC | chr5 | 56883563 | - |
| MAP3K1 | MAP3K1+6 | TTCTATGTCATGACGTGGTT | chr5 | 56898080 | + |
| MAP3K1 | MAP3K1-6 | TTGCTGGGGATCTAGTCCCA | chr5 | 56898452 | - |
| MCL1 | MCL1+1 | AACAAGGTATGCACGAGATA | chr1 | 150563774 | + |
| MCL1 | MCL1+2 | GTAGCCAAAAGTCGCCCTCC | chr1 | 150579417 | + |
| MEN1 | MEN1+1 | CTCACACTGGATCTCCGAGG | chr11 | 64799599 | + |
| MEN1 | MEN1+2 | TCTTCTCTCCAGATGTCTAC | chr11 | 64814162 | + |
| MPL | MPL+1 | TCACCCAGGCTGGCGTACCG | chr1 | 43336331 | + |
| MPL | MPL+2 | CACCCCATAGAGTTGTGACG | chr1 | 43354044 | + |
| MUTYH | MUTYH+1 | CTGTCCCTCCCGTTCGCGTG | chr1 | 45327455 | + |
| MUTYH | MUTYH+2 | CAAGGCATCCCCTACCATAA | chr1 | 45342074 | + |
| MYC | MYC+1 | GGACAATTGCTTCAGGCCGG | chr8 | 127731754 | + |
| MYC | MYC+2 | GAAAGCATGTAACCAATGTC | chr8 | 127746329 | + |
| MYCL | MYCL+1 | GATGAAGAGACTCGTTACCA | chr1 | 39887086 | + |

| | | | | | |
|---|---|---|---|---|---|
| MYCL | MYCL+2 | GGGAAGCCAATCGCAGCGCG | chr1 | 39901617 | + |
| MYCN | MYCN+1 | TGCAAGCCCGGGGGTCCAAA | chr2 | 15940283 | + |
| MYCN | MYCN+2 | AGGTGCAGGTCTCCCGACCT | chr2 | 15954908 | + |
| MYD88 | MYD88+1 | CTTTTCTTGTTGCACCTGAC | chr3 | 38128902 | + |
| MYD88 | MYD88+2 | GTCTATCTGCTGCCACGTCC | chr3 | 38143559 | + |
| NBN | NBN+1 | TCTACTCAAGCCTAATCAGT | chr8 | 89932987 | + |
| NBN | NBN-1 | GCATAGATGCTCCTTAAGTT | chr8 | 89933386 | - |
| NBN | NBN+2 | GCACAGCTATCATTCGTATA | chr8 | 89949564 | + |
| NBN | NBN-2 | TCCATCAACCTTACTCAATG | chr8 | 89949975 | - |
| NBN | NBN+3 | CCAAAAAACCATATTGTAAC | chr8 | 89968271 | + |
| NBN | NBN-3 | GAAGCAGTAACCCGGCAGTG | chr8 | 89968740 | - |
| NBN | NBN+4 | TGAAATGTGCTGCGTTAAAA | chr8 | 89984767 | + |
| NBN | NBN-4 | GACTACATTATGTGGGACGT | chr8 | 89985242 | - |
| NF2 | NF2+1 | TAGTGGTCTGGGCGACCTGA | chr22 | 29603497 | + |
| NF2 | NF2-1 | GACCCTGCCTGAGCGGAAAG | chr22 | 29603830 | - |
| NF2 | NF2+2 | ACTGGGTGCCCACGTTGACC | chr22 | 29620866 | + |
| NF2 | NF2-2 | CTGCCTCAGCGGCCCCAACA | chr22 | 29621217 | - |
| NF2 | NF2+3 | GATTTGGTGTGCCGGACTCT | chr22 | 29636777 | + |
| NF2 | NF2-3 | GCAATCAGGCTGGTAACAAT | chr22 | 29637138 | - |
| NF2 | NF2+4 | TTCCAAGTCATTGTCCCCGG | chr22 | 29654015 | + |
| NF2 | NF2-4 | ACAAGAATGTGACACTTGTA | chr22 | 29654425 | - |
| NF2 | NF2+5 | GAGAAGGAAAGCCGATTCTT | chr22 | 29668373 | + |
| NF2 | NF2-5 | AAGAAAGCAAGCTAGCTAGC | chr22 | 29668769 | - |
| NF2 | NF2+6 | CACTGAGCCCTTACGAGGGC | chr22 | 29683158 | + |
| NF2 | NF2-6 | AGAGGCTGTCAACGCATAAA | chr22 | 29683497 | - |
| NF2 | NF2+7 | CCCTGCTAGATAGCACCCCG | chr22 | 29698188 | + |
| NF2 | NF2-7 | CCGTGTTCTTGACGCCTCAA | chr22 | 29698659 | - |
| NFKBIE | NFKBIE+1 | GAGGACAAGGTTCGGAGCGC | chr6 | 44265506 | + |
| NKX2-1 | NKX2-1+1 | ACTCAAATGCTGGCGGCCTC | chr14 | 36505530 | + |
| NKX2-1 | NKX2-1+2 | GAACCCTGGGGCCGCACTGT | chr14 | 36520186 | + |
| NOTCH1 | NOTCH1+1 | GTGGTCTCCAGCCCGTAGAG | chr9 | 136493969 | + |
| NOTCH1 | NOTCH1-1 | CTGCTGGGAGAGTGACGGTG | chr9 | 136494344 | - |
| NOTCH1 | NOTCH1+2 | CAAACAAGGGCTGCGATGGA | chr9 | 136511762 | + |
| NOTCH1 | NOTCH1-2 | GCTTCCTTTCGCACAATTGT | chr9 | 136512095 | - |
| NOTCH1 | NOTCH1+3 | TGGTTTAAAATGTGCGAAGG | chr9 | 136530277 | + |
| NOTCH1 | NOTCH1-3 | CACACTCCAAACAGCCTGCG | chr9 | 136530610 | - |
| NOTCH1 | NOTCH1+4 | AACTCGGCTCCAGGCACGGG | chr9 | 136545491 | + |
| NOTCH1 | NOTCH1-4 | GCGCAGCGAAGGAACGAGCC | chr9 | 136545982 | - |
| NRAS | NRAS+1 | TTCCTCTTTTCCGTTATTAG | chr1 | 114703666 | + |
| NRAS | NRAS+2 | GTAAGCAACCATTAGGTTAG | chr1 | 114718437 | + |
| OR5L1 | OR5L1+1 | AGAAGCAATGGCCCTCCAAT | chr11 | 55809084 | + |
| OR5L1 | OR5L1+2 | GGATACAGTCACCGAGATAC | chr11 | 55820525 | + |

| | | | | | |
|---|---|---|---|---|---|
| PHOX2B | PHOX2B+1 | TCCTGCCTGTTGATCTAATG | chr4 | 41740060 | + |
| PHOX2B | PHOX2B+2 | TTGGGCTCAATTGTGGCACG | chr4 | 41754715 | + |
| PIK3R1 | PIK3R1+1 | AAACTCTCTTCCCTTCTCGC | chr5 | 68215058 | + |
| PIK3R1 | PIK3R1-1 | GCTCTGGCTCTACACGTCCC | chr5 | 68215487 | - |
| PIK3R1 | PIK3R1+2 | CAGGGTAGACAGAGGGTGCG | chr5 | 68229595 | + |
| PIK3R1 | PIK3R1-2 | GGGAAGAGCTGTACTAGCAG | chr5 | 68230118 | - |
| PIK3R1 | PIK3R1+3 | AAATGGCTCCTTAAGCTTCG | chr5 | 68243850 | + |
| PIK3R1 | PIK3R1-3 | TTTTTCTTCTACCTAACTGG | chr5 | 68244205 | - |
| PIK3R1 | PIK3R1+4 | AGAAAACATCTGACAGCACG | chr5 | 68258147 | + |
| PIK3R1 | PIK3R1-4 | AGACAGAAGGTTCAATGATC | chr5 | 68258640 | - |
| PIK3R1 | PIK3R1+5 | AAGTCACTCAGGAAATCGAG | chr5 | 68271342 | + |
| PIK3R1 | PIK3R1-5 | ATTAGCCATTGCAACTACCC | chr5 | 68271775 | - |
| PIK3R1 | PIK3R1+6 | TGGGGTTTAATATTCGTCCG | chr5 | 68287857 | + |
| PIK3R1 | PIK3R1-6 | TCACTGGCTGCTGAGCGCGC | chr5 | 68288190 | - |
| PIK3R1 | PIK3R1+7 | AAGACATAGTCTAGTAGTAG | chr5 | 68302723 | + |
| PIK3R1 | PIK3R1-7 | GGAATTACCCTAGCTCTTAC | chr5 | 68303159 | - |
| PIK3R2 | PIK3R2+1 | TGCGCTCGGGCATTTCCGCC | chr19 | 18153131 | + |
| PIK3R2 | PIK3R2+2 | GGAATCACAGCTCCCCGTAC | chr19 | 18170256 | + |
| POLD1 | POLD1+1 | GGTGAGATTAGACCCCCATT | chr19 | 50379686 | + |
| POLD1 | POLD1-1 | CCTCCTCAGCCTCGAGTGGC | chr19 | 50380080 | - |
| POLD1 | POLD1+2 | CCCTCCTGGCAGCGCACACT | chr19 | 50394260 | + |
| POLD1 | POLD1-2 | GGCAGATCCTAGATGCGGCT | chr19 | 50394775 | - |
| POLD1 | POLD1+3 | GGTCTCAATCTCCGTTCTTC | chr19 | 50406106 | + |
| POLD1 | POLD1-3 | TCAAAGAGTGAGGTCAGACG | chr19 | 50406623 | - |
| POLD1 | POLD1+4 | ACTCAACTGACTCGCCACCA | chr19 | 50419606 | + |
| POLD1 | POLD1-4 | AGACATCAAAAATACGGATG | chr19 | 50420138 | - |
| POLR2L | POLR2L+1 | AGTGCGCGGCCACGACAGCT | chr11 | 833029 | + |
| POLR2L | POLR2L+2 | AGGGGGTGGGAGGGGTCTTA | chr11 | 847728 | + |
| PTCH2 | PTCH2+1 | GCAGGACAGGTGCCCGACTC | chr1 | 44813336 | + |
| PTCH2 | PTCH2-1 | GGCCTCATCCCCTCTCCGCA | chr1 | 44813693 | - |
| PTCH2 | PTCH2+2 | CTGGCCTAGAAGGTCCCGTG | chr1 | 44827956 | + |
| PTCH2 | PTCH2-2 | CCATCCTCAGCCTGGACCTA | chr1 | 44828361 | - |
| PTCH2 | PTCH2+3 | TACACACACCTGTCGTCGGG | chr1 | 44842612 | + |
| PTCH2 | PTCH2-3 | ATGGGAGCGCCCAGATAACG | chr1 | 44842988 | - |
| RAD51C | RAD51C+1 | AAAAAAAATCGCCAATAGGT | chr17 | 58691677 | + |
| RAD51C | RAD51C-1 | CGGGAGTCGTATCCCGAGCA | chr17 | 58692018 | - |
| RAD51C | RAD51C+2 | GCAAATTTCATTGGACCATA | chr17 | 58706103 | + |
| RAD51C | RAD51C-2 | GGGCAATGCTATGTCGTCCT | chr17 | 58706595 | - |
| RAD51C | RAD51C+3 | GATTAATTGGTATAACCTAG | chr17 | 58724801 | + |
| RAD51C | RAD51C-3 | TAAAGCACTACGTATTGTGG | chr17 | 58725144 | - |
| RAD51C | RAD51C+4 | CCTCATCCACCCGCGTAGCT | chr17 | 58739887 | + |
| RAD51C | RAD51C-4 | TAGAGGTGTGGTACTACCTT | chr17 | 58740252 | - |

| | | | | | |
|---|---|---|---|---|---|
| RIT1 | RIT1+1 | ACTTTAAGTAACCCTTGATC | chr1 | 155897528 | + |
| RIT1 | RIT1+2 | CTTAAATTAACAACTTACCG | chr1 | 155911631 | + |
| RNF43 | RNF43+1 | GACAGACACAATGGGCCAAT | chr17 | 58344093 | + |
| RNF43 | RNF43-1 | GCTGTCCTGGGTGTCTGACC | chr17 | 58344588 | - |
| RNF43 | RNF43+2 | GGTTCTTGGTAAGATCGAGA | chr17 | 58358771 | + |
| RNF43 | RNF43-2 | CCTTTTTCTAAGGTACCCAC | chr17 | 58359126 | - |
| RNF43 | RNF43+3 | TCTCCATGCTAATGATAGTC | chr17 | 58373959 | + |
| RNF43 | RNF43-3 | TTAGTAAATGAGTTCGGGGA | chr17 | 58374293 | - |
| RNF43 | RNF43+4 | GTAAAGCCAATAGGTCCTAC | chr17 | 58388673 | + |
| RNF43 | RNF43-4 | ATCGTGCAGTACTAGATTAC | chr17 | 58389171 | - |
| RNF43 | RNF43+5 | TCTGGCAACACTTACGAGGC | chr17 | 58401430 | + |
| RNF43 | RNF43-5 | TAGGAAGGTAAGGTAGGATC | chr17 | 58401949 | - |
| RNF43 | RNF43+6 | TTCCTGCTTAAAACGTGCAA | chr17 | 58419493 | + |
| RNF43 | RNF43-6 | TTTAAATGCCGTTAGGGATA | chr17 | 58420009 | - |
| RPL10 | RPL10+1 | CCCGGGTTGACAAAGGAACG | chrX | 154397893 | + |
| RPL10 | RPL10+2 | CTTACAGCTCCTTTAACGTC | chrX | 154412452 | + |
| RRAS | RRAS+1 | CCTCAGGTGATCCATCCGCG | chr19 | 49634164 | + |
| RRAS | RRAS+2 | GGGCTCTTTGGGGGCGAATG | chr19 | 49650066 | + |
| SDHD | SDHD+1 | ATCAGCAAGATATTACCAAC | chr11 | 112082469 | + |
| SDHD | SDHD+2 | CAGGAGTTTAAAACCAGCGT | chr11 | 112097248 | + |
| SH2B3 | SH2B3+1 | CTCCTCTTCACAACCCTCGA | chr12 | 111401533 | + |
| SH2B3 | SH2B3-1 | TGAATATAGTGCAGGCTCGG | chr12 | 111401899 | - |
| SH2B3 | SH2B3+2 | GAAGCATTTTCTACGTTCTA | chr12 | 111416318 | + |
| SH2B3 | SH2B3-2 | ATCCCAGCACCAGCCACGCG | chr12 | 111416803 | - |
| SH2B3 | SH2B3+3 | AATGCTGCTATGAACATTCG | chr12 | 111432949 | + |
| SH2B3 | SH2B3-3 | GGAGTCCTCTCACGCAATGA | chr12 | 111433452 | - |
| SH2B3 | SH2B3+4 | TTTGATCATTTCTGGACCGT | chr12 | 111451179 | + |
| SH2B3 | SH2B3-4 | CAAAGGGAAGAGCGAGTATT | chr12 | 111451710 | - |
| SHH | SHH+1 | GGCCTTGACGCTCCTCGCAG | chr7 | 155798283 | + |
| SHH | SHH+2 | GACTCAGCCTCTGTGGACCG | chr7 | 155812947 | + |
| SMARCA4 | SMARCA4+1 | CTCCTTCCCCACTAGACCGA | chr19 | 10960333 | + |
| SMARCA4 | SMARCA4-1 | CGCGGTGACGATGGGGCAAC | chr19 | 10960672 | - |
| SMARCA4 | SMARCA4+2 | GCTGGGGGGAGGCTGCGTTA | chr19 | 10977282 | + |
| SMARCA4 | SMARCA4-2 | CACGTGAAAGGTATCGCACT | chr19 | 10977736 | - |
| SMARCA4 | SMARCA4+3 | CAGAGAGAGAAACCCAACCG | chr19 | 10993962 | + |
| SMARCA4 | SMARCA4-3 | AAAACCTAGAATATCGCTTT | chr19 | 10994321 | - |
| SMARCA4 | SMARCA4+4 | TTAGAGCTGTCCTATCCAAT | chr19 | 11003542 | + |
| SMARCA4 | SMARCA4-4 | AAAAAATCATAAGTCGGACG | chr19 | 11004021 | - |
| SMARCA4 | SMARCA4+5 | TATTGGCTGTGCCGCCTGGT | chr19 | 11018041 | + |
| SMARCA4 | SMARCA4-5 | TGCCACCCTCCAGCCACGGA | chr19 | 11018535 | - |
| SMARCA4 | SMARCA4+6 | TTAGCCTCCTCGTACTGTGC | chr19 | 11032336 | + |
| SMARCA4 | SMARCA4-6 | GTGCACACAGAACCGGAACC | chr19 | 11032860 | - |

| | | | | | |
|---|---|---|---|---|---|
| SMARCA4 | SMARCA4+7 | CACATAGGGCACATCAACTC | chr19 | 11046623 | + |
| SMARCA4 | SMARCA4-7 | TAGAGAATGACGGCACTTGA | chr19 | 11047149 | - |
| SMARCA4 | SMARCA4+8 | GCATAGGCCTTAGCAGTAAC | chr19 | 11061867 | + |
| SMARCA4 | SMARCA4-8 | CTCCTGCTGGCAGAAACGGT | chr19 | 11062332 | - |
| SMARCB1 | SMARCB1+1 | GGAAGGCTTCCCCCGTCTCG | chr22 | 23783474 | + |
| SMARCB1 | SMARCB1-1 | GTTTGCCAGGACCTGCATAG | chr22 | 23783820 | - |
| SMARCB1 | SMARCB1+2 | CTTGAGTTGATAGCTAGACC | chr22 | 23798013 | + |
| SMARCB1 | SMARCB1-2 | GGTCCCCTCTACAACCTATC | chr22 | 23798481 | - |
| SMARCB1 | SMARCB1+3 | CACAGGCCTGGAAATCGCTG | chr22 | 23816301 | + |
| SMARCB1 | SMARCB1-3 | AGCCAAACATCTCCGGATGC | chr22 | 23816685 | - |
| SMARCB1 | SMARCB1+4 | ACTCCTCTTCCAGGCGGATG | chr22 | 23834127 | + |
| SMARCB1 | SMARCB1-4 | CATACCACCTTCGTCCCCCC | chr22 | 23834604 | - |
| SOX2 | SOX2+1 | GGAAGAGCGCAGTACGGGAG | chr3 | 181711380 | + |
| SOX2 | SOX2+2 | GACAAGGAGGCGAGCCACGC | chr3 | 181726076 | + |
| SPRY1 | SPRY1+1 | CACCAATTTAGGACACGTCC | chr4 | 123393547 | + |
| SPRY1 | SPRY1+2 | ACTTGCTGCATTTGTACACG | chr4 | 123408338 | + |
| SPRY2 | SPRY2+1 | TAAGAATAAACATAGCGACT | chr13 | 80330585 | + |
| SPRY2 | SPRY2+2 | GCGGGTCTGGTTTTTTCCGG | chr13 | 80345366 | + |
| SPRY4 | SPRY4+1 | TGGGGGCAATCGGGACATAA | chr5 | 142310082 | + |
| SPRY4 | SPRY4+2 | TGCCCAGGGGTCGGAAACGG | chr5 | 142324606 | + |
| STK11 | STK11+1 | TCGCCCCAAAGGGGGATGCG | chr19 | 1205322 | + |
| STK11 | STK11-1 | AGGACGACGTGCCCACCCGT | chr19 | 1205714 | - |
| STK11 | STK11+2 | CACCCACTGAGACCGGCTCT | chr19 | 1222517 | + |
| STK11 | STK11-2 | CTGGGACCAGGCCGTGCAAG | chr19 | 1222878 | - |
| STK11 | STK11+3 | TTCCGCCCACACGCGACAGC | chr19 | 1237168 | + |
| STK11 | STK11-3 | GTGAGGTCGGGCGAGGACGA | chr19 | 1237553 | - |
| TBX3 | TBX3+1 | GTGACACAGCTAAGTTTTCG | chr12 | 114669533 | + |
| TBX3 | TBX3+2 | AGAAGGTCGGGCTGCGCTGT | chr12 | 114684188 | + |
| TFE3 | TFE3+1 | TAGCACAGAAAAGACGTTAG | chrX | 49025079 | + |
| TFE3 | TFE3+2 | GCCGCATGAGACATGACGCC | chrX | 49043212 | + |
| TMPRSS2 | TMPRSS2+1 | TGAGGCAGGAAAATCGAGTC | chr21 | 41461344 | + |
| TMPRSS2 | TMPRSS2-1 | TGGTTTGTTCAATACACTCC | chr21 | 41461877 | - |
| TMPRSS2 | TMPRSS2+2 | TGAGCCAGCCCTGCCGGGAT | chr21 | 41475893 | + |
| TMPRSS2 | TMPRSS2-2 | GCCCTGGCAGCCACCTAACG | chr21 | 41476246 | - |
| TMPRSS2 | TMPRSS2+3 | AATTGGTGCTACTCAATAAC | chr21 | 41492485 | + |
| TMPRSS2 | TMPRSS2-3 | AAGTCAGCATCTCCAGACGG | chr21 | 41492853 | - |
| TMPRSS2 | TMPRSS2+4 | CCAACTGGCTAGGGACTCGC | chr21 | 41507774 | + |
| TMPRSS2 | TMPRSS2-4 | CTGAGATTAAAGCGAGAGCC | chr21 | 41508192 | - |
| TP53 | TP53+1 | TTCCTGCACCCGCCCTACCA | chr17 | 7657641 | + |
| TP53 | TP53-1 | TCACTGTACAAAAGGGACGT | chr17 | 7658007 | - |
| TP53 | TP53+2 | CATCTCATGGGGTTATAGGG | chr17 | 7674734 | + |
| TP53 | TP53-2 | GAGCGCTGCTCAGATAGCGA | chr17 | 7675073 | - |

| | | | | | |
|---|---|---|---|---|---|
| TP53 | TP53+3 | GTAAGGATAACAACGGGGCA | chr17 | 7689322 | + |
| TP53 | TP53-3 | CACTTTTTCCATCTCCACGA | chr17 | 7689771 | - |
| VEGFA | VEGFA+1 | GCTGGTAGCGGGGAGGATCG | chr6 | 43770191 | + |
| VEGFA | VEGFA+2 | GGGAGTGGGAGTTGTATTGG | chr6 | 43787353 | + |
| VHL | VHL+1 | AAAAATTCAGTTAGTCCACC | chr3 | 10141257 | + |
| VHL | VHL+2 | ACACATACCCAGTAGTCCAT | chr3 | 10155938 | + |
| ZNF703 | ZNF703+1 | CAAAGGACCAACTTCGCCTC | chr8 | 37694611 | + |
| ZNF703 | ZNF703+2 | CCCACTGAGCCCTATCTACC | chr8 | 37709182 | + |

** SMARCB1-4 sgRNA was omitted accidentally in experiments
